# Optimization of *Paenibacillus* sp. NMA1017 Application as a Biocontrol Agent for *Phytophthora tropicalis* and *Moniliophthora roreri* in Cacao-Growing Fields in Chiapas, Mexico

**DOI:** 10.3390/plants12122336

**Published:** 2023-06-15

**Authors:** Irene Gómez-de la Cruz, Belén Chávez-Ramírez, Carlos Hugo Avendaño-Arrazate, Yolanda Elizabeth Morales-García, Jesús Muñoz-Rojas, Paulina Estrada-de los Santos

**Affiliations:** 1 Laboratorio de Biotecnología Microbiana, Escuela Nacional de Ciencias Biológicas, Instituto Politécnico Nacional, Prolongación Carpio y Plan de Ayala s/n, Col. Santo Tomas, Alcaldía Miguel Hidalgo 11340, Ciudad de México, Mexico; igomezdl@ipn.mx; 2 Laboratorio de Fitopatología, Escuela Nacional de Ciencias Biológicas, Instituto Politécnico Nacional, Prolongación Carpio y Plan de Ayala s/n, Col. Santo Tomas, Alcaldía Miguel Hidalgo 11340, Ciudad de México, Mexico; bchavezra@ipn.mx; 3 Campo Experimental Rosario Izapa, Instituto Nacional de Investigaciones Forestales, Agrícolas y Pecuarias, Km 18, Carretera Tapachula-Cacahoatán, Tuxtla Chico 30870, Chiapas, Mexico; avendano.carlos@inifap.gob.mx; 4 Ecology and Survival of Microoganisms Gropu, Laboratorio de Ecología Microbiana, Centro de Investigaciones en Ciencias Microbiológicas, Instituto de Ciencias, Benemérita Universidad Autónoma de Puebla, San Manuel, Puebla 72570, Puebla, Mexico; yolanda.moralesg@correo.buap.mx

**Keywords:** moniliasis, black pod rot, biocontrol, *Phytophthora*, *Moniliophthora roreri*, *Theobroma cacao*

## Abstract

In Mexico, cacao production is endangered by pathogenic fungi, such as *Phytophthora* spp. and *Moniliophthora rorei,* that cause black pod rot and moniliasis, respectively. In this study the biocontrol agent *Paenibacillus* sp. NMA1017 was tested in cacao fields against the previous diseases. The treatments applied were shade management, inoculation of the bacterial strain with or without an adherent, and use of chemical control. The statistical analysis showed that the incidence of black pod rot in tagged cacao trees diminished when the bacterium was applied (reduction of 44.24 to 19.11%). The same result was observed with moniliasis when the pods were tagged (reduction of 66.6 to 27%). The use of *Paenibacillus* sp. NMA1017 with an integrated management might be a solution to cacao diseases and to having a sustainable production of cacao in Mexico.

## 1. Introduction

In 2020, the world cacao production was valued at 7.5 million US dollars. The largest producer was Ivory Coast, with 2.2 million tons and a yield of 460.6 kg/ha [1]. In the world, Mexico is the 14th largest producer of cacao, with 29,429 tons in a harvested area of 58,598 ha and a yield of 502.2 kg/ha [1]. Mexico produces the Criollo cacao, which possess a higher quality over others in the cacao market [2]. Nevertheless, the production is small; only 7% of the cacao production is Criollo, which might be due to different factors, but it is especially due to diseases [3]. Among the previous diseases, moniliasis caused by the fungi *Moniliophthora roreri* and black pod rot produced by several species of the oomycete *Phytophthora* are devastating in Mexico [4]. The latter is largely distributed in cacao producer countries, while the former is only found in America. Without adequate management, the losses can reach up to 70 to 100% [5]. To recognize these diseases, the symptoms and signals for moniliasis in 1–2-month-old pods are deformations, necrosis, and decomposition, which seem to be related to hypertrophy and hyperplasia. Infected fruits that reach later stages show irregular pigmentation or dark, oily spots, and in a period of three to eight days, the fruits are covered with *M. roreri* spores. Internal tissues can form an aqueous mass, which is the result of the cacao pod decomposition [6]. In black pod rot, the infection starts in the base pod or the apex, with brown, round spots that increase in size by one to two cm per day. The disease spreads fast in the pod, covering it in 15 days and producing white spores that are the source of new infections. When the pathogen attacks the roots, it hinders the intake of nutrients and water, causing the death of the cacao tree [7].

These phytosanitary problems are managed with chemical products, such as those containing copper derivatives. Using copper derivatives is allowed for the control of phytopathogens in organic crops; however, given that copper is immobilised in soil, it modifies the soil pH, it accumulates, and it provokes phytotoxicity and phytopathogen resistance. Moreover, it diminishes the diversity of microorganisms in the soil and is even related to an increase in diseases, such as Alzheimer’s [8,9]. Additionally, this element might be related to growth problems, brain disfunction, and metabolic activity in the human body [10].

Nevertheless, the socioeconomic conditions of Mexican cacao farmers impede getting these chemical compounds or getting better controlling options for cacao diseases. However, the interest of conserving cacao Criollo varieties has led to considering biocontrol alternatives, which are environmentally safe. Previously, our research group applied *Paenibacillus* sp. NMA1017 in semi-controlled experiments in cacao fields, which showed a reduction in the disease incidence (86 to 33%) and infection (68 to 6%) of black pod rot [11]. In this study, the capacity of *Paenibacillus* sp. NMA1017 to diminish the incidences and severities of moniliasis and black pod rot was tested in a field with cacao Criollo (Carmelo variety) grown in a Mexican experimental zone that imitated cacao farmers’ agricultural practices.

## 2. Results

### 2.1. First Bioassay: Incidence of Moniliasis and Black Pod Rot in Tagged Pods

**Figure 1:**
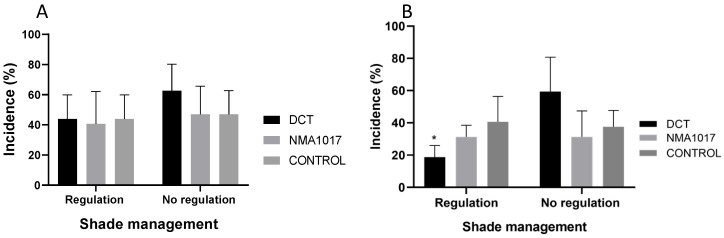
Incidence of (**A**) black pod rot and (**B**) moniliasis (*Moniliphthora roreri*) in cacao pods under different treatments in the first bioassay. The treatments were regulation of shade and no regulation of shade. DCT, dicopper chloride trihydroxide. NMA1017, application of *Paenibacillus* sp. NMA1017. CONTROL, negative control with water. The asterisk indicates a statistical difference when compared with the negative control.

In this first study, the management of shade was evaluated. The analysis of variance for the black pod rot incidence showed that the management of shade (*p* = 0.2118) and the applied treatments (*p* = 0.5382) had no significative statistical effects on this disease. However, in the moniliasis incidence, there was a significative statistical effect with shade managing (*p* = 0.0419) and the treatments applied (*p* = 0.0095). The treatment with dicopper chloride trihydroxide + regulated shade resulted in a lower incidence of moniliasis (18.7%), compared to the control, which was 40.6%. The incidence of moniliasis in pods treated with *Paenibacillus* sp. NMA1017 were similar, both with the management of shade and with no management (around 30%) (Figure 1). All infected pods were affected with moniliasis and black pod rot at a severity scale of 5, with more than 80% internal damage.

### 2.2. Second Bioassay: Incidence of Moniliasis and Black Pod Rot in Tagged Plants

**Figure 2:**
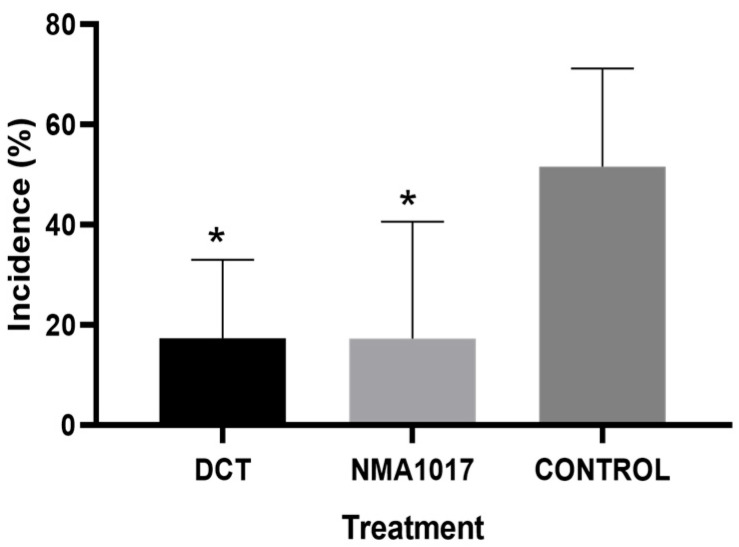
Incidence of black pod rot in tagged cacao plants under different treatments in the second bioassay. DCT, dicopper chloride trihydroxide. NMA1017, application of *Paenibacillus* sp. NMA1017. CONTROL, negative control with water. The asterisks indicate statistical differences when compared with the negative control analyzed with the Tukey test.

To improve the results from the first bioassay, in the second bioassay, some changes were introduced. Performing the experiment with regulated shade, adding an adherent, and applying the treatments every two weeks were the new parameters to consider. The analysis of variance showed that there was no statistical difference in moniliasis with any of the treatments (*p* = 0.0737). However, there was a statistical difference in the black pod rot disease (*p* = 0.021). The Tukey test indicated statistical differences in the treatments of dicopper chloride trihydroxide (*p* = 0.230) and *Paenibacillus* sp. NMA1017 (*p* = 0.0228), compared to the control (Figure 2).

**Figure 3:**
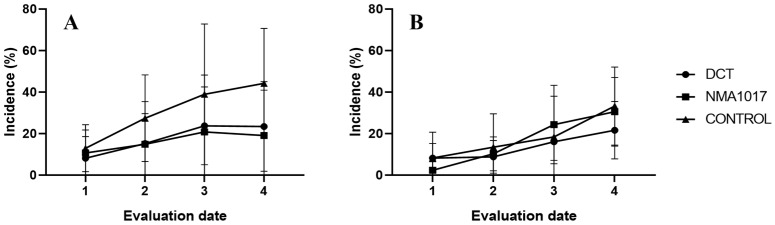
Incidence of moniliasis and black pod rot in tagged cacao trees under different treatments through time. (**A**) Incidence curve of moniliasis (*Moniliophthora roreri*). (**B**) Incidence curve of black pod rot (*Phytophthora tropicalis*). DCT, dicopper chloride trihydroxide. NMA1017, application of *Paenibacillus* sp. NMA1017. CONTROL, negative control with water. The evaluation dates were every 15 days.

Moniliasis incidence was present in all treatments, with a 21.6% incidence for dicopper chloride trihydroxide treatment, 30.5% incidence for *Paenibacillus* sp. NMA1017 treatment, and 33.3% incidence for the control with water (Figure 3A). The incidence of black pod rot was 19.11% for *Paenibacillus* sp. NMA1017, 21.6% for dicopper chloride trihydroxide treatment, and 44.24% for the control (Figure 3B).

### 2.3. Second Bioassay: Incidence of Moniliasis and Black Pod Rot in Tagged Pods

**Figure 4:**
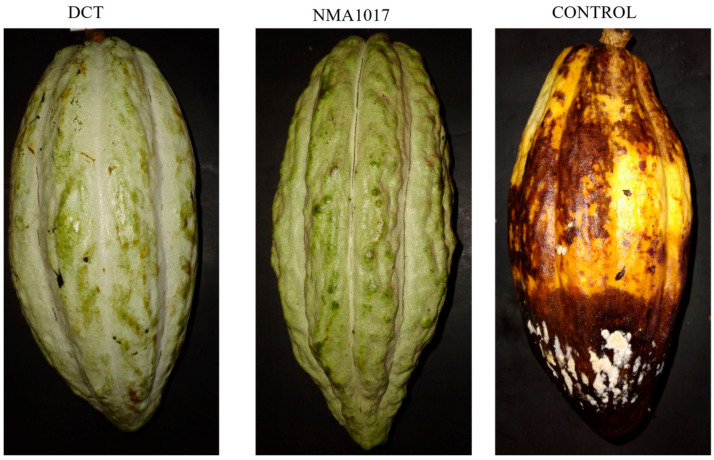
Incidence of moniliasis (*Moniliophthora roreri*) in tagged cacao pods under different treatments in the second bioassay. The treatments were: DCT, dicopper chloride trihydroxide; NMA1017, application of *Paenibacillus* sp. NMA1017; and CONTROL, negative control with water.

**Figure 5:**
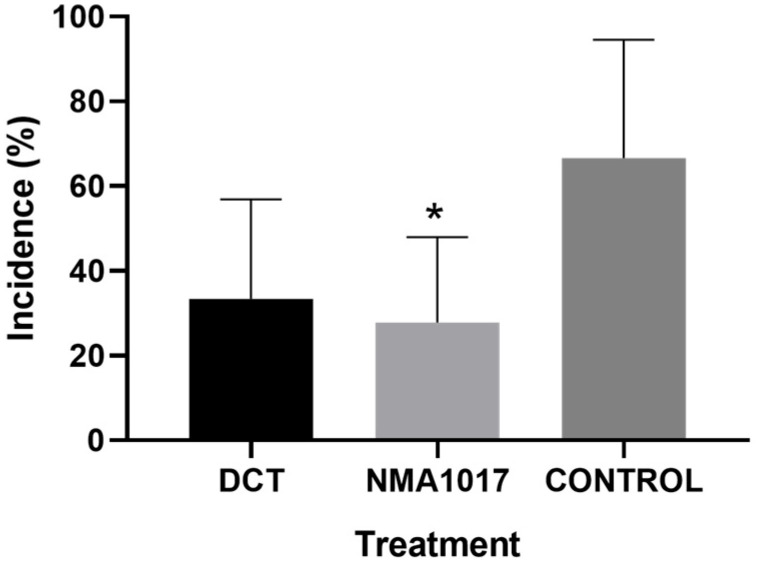
Graphical incidence of moniliasis (*Moniliophthora roreri*) in tagged cacao pods under different treatments in the second bioassay. DCT, dicopper chloride trihydroxide. NMA1017, application of *Paenibacillus* sp. NMA1017. CONTROL, negative control with water. The asterisk indicates statistical differences when compared with the negative control.

The analysis of variance showed that there were no statistical differences in the incidences of black pod rot (*p* = 0.2593). However, there were differences in the treatment for moniliasis (*p* = 0.0281). The Tukey analysis indicated statistical differences between the incidence of moniliasis for pods treated with *Paenibacillus* sp. NMA1017 and the control with water (*p* = 0.0342). The incidence of moniliasis in the treatment with the bacteria was 27% (Figure 4), the chemical control was 33.3%, and the control with water was 66.6% (Figure 5).

**Figure 6:**
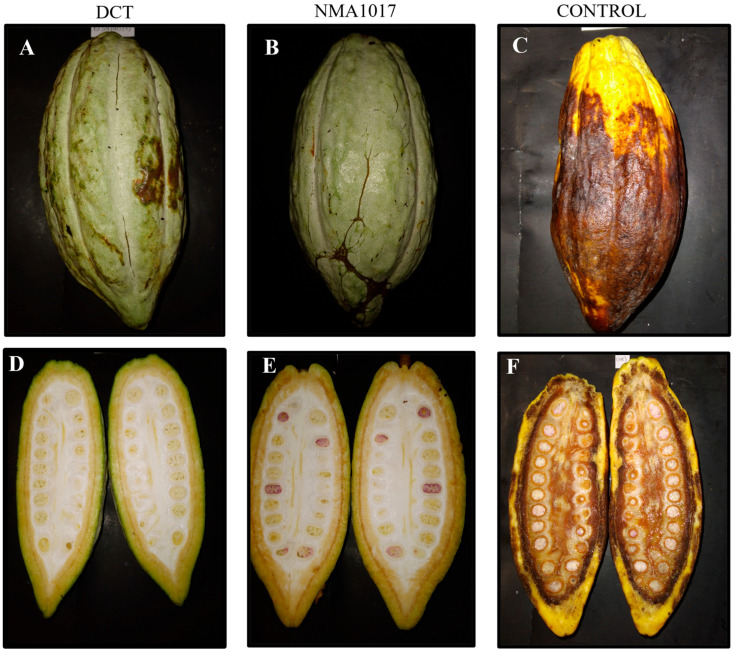
External (**A**–**C**) and internal (**D**–**F**) severities of moniliasis (*Moniliophthora roreri*) in tagged cacao pods. The treatments were: DCT, dicopper chloride trihydroxide; NMA1017, application of *Paenibacillus* sp. NMA1017; and CONTROL, negative control with water.

Moreover, there were statistical differences in the external (*p* ≤ 0.0001) and internal (*p* = 0.0026) severities of moniliasis (Figure 6).

**Figure 7:**
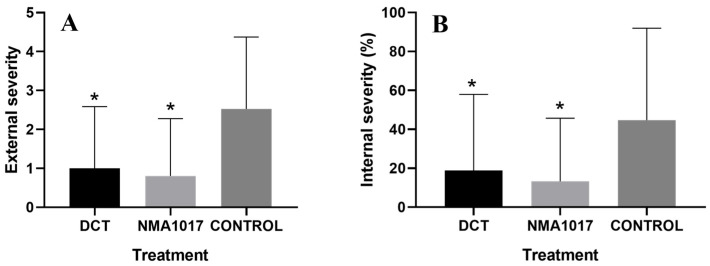
Graphic of external (**A**) and internal (**B**) severities of moniliasis (*Moniliophthora roreri*) in tagged cacao pods. The treatments were: DCT, dicopper chloride trihydroxide; NMA1017, application of *Paenibacillus* sp. NMA1017; and CONTROL, negative control with water. The asterisk indicates statistical differences when compared with the negative control.

The comparison of media in the Tukey analysis showed statistical differences in the external severities in the treatments with *Paenibacillus* sp. NMA1017 (*p* ≤ 0.0001) and dicopper chloride trihydroxide (*p* = 0.0004), compared to the control. The treatment with the biological agent had the lower external severity at 0.8% (between 0 and 1% damage), the chemical treatment severity was 1 (between 1 and 20% damage), and the control with water was 2.5 (between 21 and 40% damage) (Figure 7A). The internal severity with the comparison of media from the Tukey analysis showed statistical differences when comparing the chemical treatment (*p* = 0.0200) and *Paenibacillus* sp. NMA1017 (*p* = 0.0035) with the control. The biological treatment resulted in a lower internal severity (Figure 7B) at 13.3%, then the chemical treatment at 18.8% and the control with water at 44.6%.

### 2.4. Phytopathogens and Biocontrol Agent Isolation

In each evaluation, the two phytopathogens were isolated in at least one pod from each treatment. The phytopathogens were identified by colony morphology and microscopic features. The isolation of the bacterium NMA1017 was easy to perform, since it has a very particular colonial morphology that produces a large amount of extracellular polymeric substances (EPS). The bacteria were isolated in PDA along the experiment, even 15 days after the experiments was completed. This result corroborated the presence of both the phytopathogens and the biocontrol agent.

### 2.5. Trapping of Phytopathogen Spores

**Figure 8:**
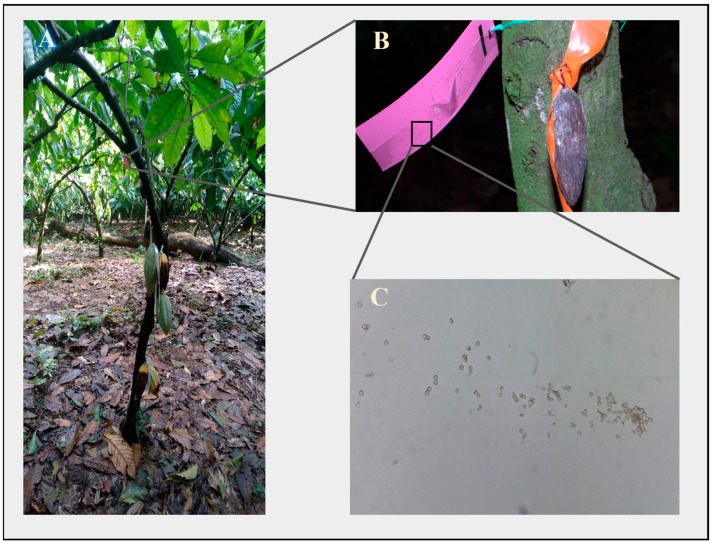
Cacao tree (**A**) with a trapping device (**B**) and *Moniliophthora roreri* spores (**C**) observed at 10×.

During the whole experiment, the traps were removed from the trees and then observed under a microscope. The traps contained spores from *M. roreri*, *P. tropicalis,* and other phytopathogens, such as *Colletrotrichum gloeosporioides* (Figure 8). However, the spores were uncountable.

Nevertheless, this assay established that the phytopathogens were in the environment and were able to cause disease, as many pods were found infected with both diseases.

## 3. Discussion

Reduced production of cacao is one of the main problems that cacao farmers face in Mexico. This is due to little or no management of plantations, advanced age and/or low scholarship of the farmers (that lead to the limitation of applying new technologies), and the presence of diseases and pests [3].

Moniliasis and black pod rot are the main problematic diseases in cacao growing in Mexico. The chemical control is optional, but the high environmental and economic costs make it unaffordable. Therefore, it is advisable to consider an integrated management that embraces cultural, chemical, biological, and genetic controls [12]. The cultural control includes the management of shade, which reduces the presence of phytopathogenic fungi in the culture. In this study, the shade management had statistical differences in the moniliasis incidence, where it was lower in the area with controlled shade. Under the condition with regulated shade, the incidence of moniliasis was lower in the chemical control and the application of the biological control, but it was not the case with the negative control. These results are different from Beer et al. [13], who indicated that increasing the shade had a positive correlation with the incidence of black pod rot; however, the incidence of moniliasis was not increased by this condition, which suggests that other factors, such as temperature or vectors, helped the pod infection. Shade regulation had an impact on humidity. The more shade there was, the more humidity there was, resulting in more phytopathogenic problems, which was not the case for moniliasis in this study.

Other than the shade, the chemical control, although expensive for Mexican farmers, was also included in the integrated management. Therefore, dicopper chloride trihydroxide was added as a positive control, since it has been proven to protect pods from moniliasis and black pod rot [4,6,14,15]. In the first assay, the chemical control with regulated shade diminished the incidence of moniliasis to 21.9%, but the bacterial application was only diminished to 9.3%. A reason for this result might be the heavy rain in the area when the experiment took place, likely washing off the bacteria. Therefore, and considering the previous results, an adherent was included. Adherents are important for improving the effectiveness of fungicides [16]. With this, changes in the incidences of both moniliasis and black pod rot were further analyzed.

In the second bioassay, when analyzing tagged cacao trees, there were no statistical differences among the treatments when moniliasis was studied. This might be due to the period of time when the experiment was performed (May; rain season had not started yet). The moniliasis cycle starts when the humidity is low; at this time, millions of conidia are produced and disseminated by the wind, establishing in the surfaces of the leaves and pods. Then, with humidity, the conidia germinate and penetrate the plant epidermis. *M. roreri* has an infection phase that lasts for seven weeks. After that, there is an abundance of sporulation for one week that later covers the pods with mycelia [17]; therefore, the presence *of M. roreri* might be difficult to eradicate but might be possible when the biocontrol agent is applied continuously.

Nevertheless, there were statistical differences in the black pod rot disease. The lowest incidence for that disease was observed with the application of the strain NMA1017. Previously, we demonstrated that this bacterium could reduce black pod rot incidence in a semi-controlled experiment (86 to 33%) [8]. In the present study, where integrated management was included, the incidence was lessened from 44.24% (control with water) to 19.11% with the bacterial strain, resulting in a behavior of pod protection that was similar to the behavior observed in our previous study.

When the experiment was focused on tagged pods, the statistical differences were observed in moniliasis but not in black pod rot. The incidence of moniliasis was reduced from 66.6% (in the control with water) to 27% with the bacterial inoculation. Moreover, the external severity in pods with moniliasis showed a reduction of 0.8 (in a scale where 0 represents healthy pods and 4 represents pods that are 100% damaged).

There are few reports on the effect of bacteria on moniliasis or black pod rot in cacao. Some examples are in Colombia, where the use of *Bacillus* sp. reduced the incidence of moniliasis by 13.5% and *Trichoderma* by 28% [18]. *Bacillus amiloliquefaciens* was found to reduce the incidence of *Phytophthora* sp. by 30% in Ghana [19]. Although the study of the biocontrol of black pod rot and moniliasis is growing in America, more work has been performed in Africa because of the presence of *Phytophthora megakarya*, a very aggressive species that attacks cacao [20]. In Mexico there are no other studies of biocontrol on black pod rot or moniliasis; therefore, the application of *Paenibacillus* sp. NMA1017 in this study diminishing the incidences and internal and external severities of moniliasis and black pod rot in a cacao field is important. The use of this bacterium, not as a stand-alone solution but with integrated management strategies, can be proposed for an organic treatment that controls moniliasis and black pod rot in Mexico. It is recommended that the biocontrol uses locally isolated fungal antagonists. However, strain NMA1017 was isolated in the center of Mexico from an agave crop [21]. This bacterium was even found on the cacao surface 15 days after the experiment was completed or even in the cacao field soil, suggesting its capability to stay in the environment for some period of time. This ability might be due to the preservation for the excessive production of extrapolymeric substances. This colony morphology was essential to recognize the bacterium in agar plates.

Thus, the results of this study offer optimism for the use of biological control agents for diseases in cacao, such as black pod rot and moniliasis, along with an integrated management strategy; it might allow us to have a sustainable production of cacao in Mexico.

## 4. Materials and Methods

### 4.1. Biocontrol Agent Growth

*Paenibacillus* sp. NMA1017 was previously isolated from *Opuntia ficus-indica* (L.) Mill. Barbary fig in Mexico City [21] and tested for biocontrol in cacao plants grown in the south of Mexico [8]. The strain NMA1017 was routinely grown in potato dextrose agar (PDA) at 30 °C for 24–48 h. The bacterial growth was used to obtain a cellular suspension in 5 mL of MgSO_4_-7H_2_O 10 mM, with an approximate optical density DO_600 nm_ of 0.5, to inoculate 500 mL of potato dextrose broth (PDB) (~6 × 10^8^ CFU/mL). The bacterium was incubated 72 h at 30 °C and 120 rpm. At the end of the incubation period, the cellular growth was 1 × 10^10^ CFU/mL, and it was diluted to 1 × 10^8^ CFU/milk. The diluted solution was used to spray cacao plants, starting at the base of the trunk, then the branches and the pods. Each plant was inoculated with 100 mL of the biological agent. The application of the biocontrol agent was carried out using a 3 L backpack fumigator (Truper).

### 4.2. Field Location and Features

The experimental field was located at the Instituto Nacional de Investigaciones Forestales, Agrícolas y Pecuarias (INIFAP) in Campo Experimental Rosario Izapa (CERI) in Tuxtla Chico, Chiapas, México (14°58′28″ N, 92°09′20″ W). The average annual temperature was 25.9 °C, the average annual rainfall was 3204.6 mm, and the relative humidity was 70% [22] The field contained 10 furrows and 50 plants per furrow. The distance between furrows was 3 m, and there were 3 m between plants. For 5 furrows, the shade was regulated (shade tree pruning to maintain 50% shade), and for the other 5 furrows, the shade was not regulated. The cacao plants were 8 years old and belonged to the Criollo Carmelo variety.

### 4.3. First Biocontrol Assay

This experiment was established under a factorial design, 2 × 3, and was completely random, where the evaluation factors were (a) regulated shade (level 1, regulated up to 50%; level 2, no shade regulation, meaning 74%) and (b) treatments (level 1, *Paenibacillus* sp. NMA1017; level 2, negative control using water; level 3, positive control using dicopper chloride trihydroxide (200 g/L). The experimental units were 8 cacao pods between 60 and 90 days old and were 12 to 15 cm long. All cacao pods were tagged. The experiment included 4 repetitions per treatment (total of 32 pods per treatment). The repetitions were randomly established in each level and according to the shade management (level 1, furrows with plants and regulated shade; level 2, furrows with plants and no shade regulation). Before starting the experiment, all diseased pods were removed. The first application of the biocontrol was on October 15, 2020. After that, 4 more subsequent applications were performed every week. The incidence of moniliasis and black pod rot in tagged pods were determined on the fourth week after the first application.

### 4.4. Second Biocontrol Assay

The experiment was similar to the first bioassay. The main differences were using only regulated shade (50%), adding the adherent Li 700^®^ 100 cc./Hl. to all treatments, and the application of the treatments every two weeks. The composition of Li 700^®^ included methylacetic acid, phosphatidylcholine, alkyl polyoxyethylene ether: 80%, and other ingredients (property of non-hazardous): 20%.

### 4.5. Whole-Plant Experiments

The experiment was completely random, with three treatments: (a) *Paenibacillus* sp. NMA1017 (~1 × 10^9^ CFU/mL), (b) dicopper chloride trihydroxide (200 g/L), and (c) water as negative control. Seven plants were tagged randomly for each treatment. The plants harbored pods in different developmental stages. The diseased pods were also removed before starting the experiment. The incidences of moniliasis and black pod rot were obtained from the tagged plants at the end of the experiment.

### 4.6. Tagged-Pod Experiments

Additionally, the same treatments applied to whole plants were carried out with 30 to 60 day-old and 6 to 10 cm long pods selected at this stage, due to the susceptibility to the pathogens. Six cacao pods were used as the experimental units, and there were six repetitions per treatment (total of 36 cacao pods per treatment).

The first application started on 27 May 2021, with 4 applications every 15 days. Additionally, every 15 days, the number of diseased pods was recorded, and the internal and external severity was determined. This continued until day 100 ± 10 before the physiological maturity was reached.

### 4.7. Incidence and Severity Determination

The incidences of moniliasis and black pod rot were determined by considering the tagged sick pods and the total pods in the cacao trees. With these results, the average incidence curves were built for each disease. The external and internal severities of moniliasis were performed according to Phillips-Mora et al. [23]. In the case of black pod rot, the incidence was carried out according to Chavez-Ramirez et al. [11]. An analysis of variance was performed with GraphPad Prism 8.0.1.

### 4.8. Phytopathogens and Biocontrol Agent Isolation

To follow the Koch’s postulates, the microorganisms were isolated from diseased cacao pods using PDA. In each evaluation, a pod with moniliasis or black pod rot symptoms and signs were taken and washed with water and soap. One cm^2^ pieces were cut, and the surfaces were sterilized with 3% sodium hypochlorite and washed with sterile water 5 times. Then, the pieces were cut (0.5 cm^2^) and placed on the PDA. The plates were incubated at 30 °C for 5 to 15 days, depending on the phytopathogen growth rate. Macro- and microscopic features for each phytopathogen were recorded and compared to the literature to identify them. At the same time, the biological agent was also isolated; for this, before each treatment application, three pods inoculated previously with the strain NMA1017 were selected randomly. A sterile cotton swab was used to pass on the pod surface. Then, the cotton swab was streaked on the PDA. The plates were incubated at 30 °C for 24 h. Macro- and microscopic morphologies were determined by comparing them with the original strain to determine the identification as strain NMA1017.

### 4.9. Trapping of Phytopathogens Spores

In order to establish that the phytopathogens were naturally in the cacao field, traps with transparent Scotch tape were placed in the cacao trees. Three plastic rectangles (3 × 12 cm) were positioned in the upper, middle, and lower parts of 10 cacao trees. The rectangles were covered with adhesive tape and left for 15 days. Then, the tape was removed before the application of the biocontrol agent and then a new tape was placed right after the bacterial application. Finally, the adhesive tape was placed on a slide that contained a drop of lactophenol cotton blue stain. The slides were observed with an optic microscope to identify the phytopathogens spores.

### 4.10. Variance Analysis

The data obtained from the incidences of moniliasis and black pod rot in the first bioassay were statistically analyzed with two-way variance analysis. The data of incidence in tagged plants and the severity of the diseases in the tagged pods in the second bioassay were analyzed with one-way variance analysis; then, Tukey’s comparison means were performed with GraphPad Prism v8.0.1.

## 5. Conclusions

The production of cacao in Mexico is at risk, due to the presence of phytopathogens such as *Phytopththora* spp. and *Moniliophthora rorei*. The use of chemical pesticides to treat the diseases provoked by these microorganisms is an option that should not be used anymore. Therefore, the alternative of using biocontrol agents instead of chemical control and combining them with integrated management in cacao production is promising for cacao Criollo in Mexico and for the manufacturing of an organic product.
